# Epidemiological Trends in Patients Undergoing Mitral Valve Transcatheter Edge-to-Edge Repair over the Last Decade: Functional vs. Structural Mitral Regurgitation

**DOI:** 10.3390/jcm11051422

**Published:** 2022-03-04

**Authors:** Leonhard Schneider, Nicoleta Nita, Tilman Dahme, Sinisa Markovic, Mirjam Keßler, Wolfang Rottbauer, Marijana Tadic

**Affiliations:** Klinik für Innere Medizin II, Universitätsklinikum Ulm, Albert-Einstein Allee 23, 89081 Ulm, Germany; leonhard.schneider@uniklinik-ulm.com (L.S.); nicoleta.nita@uniklinik-ulm.com (N.N.); tilman.dahme@uniklinik-ulm.com (T.D.); sinisa.markovic@uniklini-ul.com (S.M.); mirjam.kessler@uniklini-ulm.com (M.K.); wolfang.rottbauer@uniklinik-ulm.com (W.R.)

**Keywords:** mitral regurgitation, interventional mitral valve repair, epidemiology

## Abstract

Objective: We aimed to investigate the demographic, clinical and hemodynamic characteristics of patients who underwent percutaneous mitral valve (MV) repair over the last decade, as well as to determine the potential changes in trends of these parameters among patients with structural and functional MR (SMR and FMR). Methodology: We analyzed all patients who underwent interventional MV repair in our institution between January 2010 and March 2021. Our study included both SMR and FMR patients. All data were obtained from a local registry. Results: Nine hundred and seventeen patients (357 SMR patients and 563 FMR patients) were involved in this study. We did not find significant differences in demographical, clinical and hemodynamic characteristics among SMR and FMR patients. Left ventricular remodeling and systolic dysfunction were more pronounced in FMR patients. Systemic vascular resistance was the only hemodynamic parameter that differed between SMR and FMR patients; it was higher in SMR group. An evaluation of the trend between the first and last five years of our experience revealed that the number of patients treated with this technique is constantly increasing, but that this is more pronounced in SMR patients. It was also found that the operative risk of SMR and FMR patients was significantly higher in the first five years. Additionally, our results showed change in medical therapy in MR patients over the last decade in terms of increased use of angiotensin II receptor blockers and the introduction of angiotensin receptor II blocker-neprilysin inhibitor. Conclusion: SMR and FMR patients who underwent interventional MV repair have similar clinical and hemodynamic characteristics. The percentage of SMR patients increased more significantly than FMR patients over the last five years.

## 1. Introduction

Mitral regurgitation (MR) represents the most frequent valvular heart disease; its prevalence is constantly rising [1,2]. The main classification of MR etiology considers primary–structural MR (SMR) and secondary–functional MR (FMR). Surgical intervention was the only therapeutic option for symptomatic patients with severe MR, mainly SMR, for a long time. Transcatheter edge-to-edge repair (TEER) of the mitral valve (MV) caused a revolution in MR treatment when the MitraClip system (Abbott) was approved in Europe in 2008 for both indications, i.e., SMR and FMR, whereas it was approved in 2013 in the United States and only for SMR [3]. The treatment was primarily intended for FMR patients with very high operative risk, but it was soon adopted as an efficient therapeutic option in SMR patients with unacceptable operative risk [4,5]. More than one decade later, a new system for MV TEER, the PASCAL device (Edwards), was approved in Europe, but still not in the US. The first published data regarding the PASCAL system revealed that MR severity, irrespective of etiology, was significantly reduced, together with significant improvements in functional status, exercise capacity, and quality of life [6]. 

The number of MR patients treated with TEER is rapidly increasing, despite controversial results that were almost simultaneously published in 2018 [7,8], namely, the COAPT trial showed significant improvement in survival and reduction in hospital readmission due to heart failure after treatment with the MitraClip [7], whereas the MITRA-FR trial revealed discouraging results that showed no difference in outcome between patients treated with MitraClip or guideline-directed medical therapy [8]. There are many differences between these trials, starting from the inclusion criteria and definition of MR severity, concomitant diseases and therapy, the experience of interventional cardiologists involved in these procedures, to the observed outcomes.

Over more than one decade, our knowledge about MV repair has significantly increased; however, most published studies have been focused on the outcomes of MR patients after this procedure [4,5]. The demographic and clinical characteristics of SMR and FMR patients who underwent this procedure have been significantly less investigated. Considering the fact that SMR and FMR are two entities with substantially different etiologies, one would assume that these patients have various preprocedural characteristics that may have significant impacts on outcomes. Furthermore, it remains unknown if these characteristics changed over the last decade in both groups of MR patients, i.e., SMR and FMR. 

The aim of this study is to evaluate the demographic and clinical characteristics in a large population of patients who underwent MV TEER, and to investigate potential differences in preprocedural factors in SMR and FMR patients in the periods of 2010–2015 and 2016–2021 that may have affected decision making and outcomes in this population. 

## 2. Methodology

This is an observational retrospective study that included 917 patients who underwent MV TEER from January 2010 to March 2021 at our institution. All included patients had symptomatic, high-grade mitral regurgitation diagnosed by transthoracic and transesophageal echocardiography. The interdisciplinary heart team made decisions for interventional MV repair in accordance with the current guidelines on valvular heart disease [1]. Preprocedural transoesophageal and transthoracic echocardiography and invasive hemodynamic measurement by cardiac catheterization were performed prior to the procedures. According to the etiology of MR, all patients were divided into two groups: patients with primary (structural) MR (SMR) and patients with secondary (functional) MR (FMR) [2]. The etiology was determined by the Carpentier’s classification, and severity of MR was assessed according to the European recommendations [2].

Risk factors for surgical repair of the MV were evaluated prospectively using the European System for Cardiac Operative Risk Evaluation (EuroSCORE II) system [9]. MV TEER was performed in a hybrid catheterization laboratory under general anesthesia. MitraClip and PASCAL were used for MV repair, and they were implanted under fluoroscopic and echocardiographic guidance.

Clinical and laboratory data, as well as concomitant diseases and medical therapy, were taken from the medical record of each patient. ICD 9 and 10 classifications of disease were used for determination of existing comorbidities. All subjects were participants of the prospective MiTra ULM registry. All patients gave written informed consent for retrospective and prospective data collection. The local ethical committee approved the research protocol.

### Statistical Analysis

Continuous variables were presented as mean ± standard deviation and were compared by the Student’s *t*-test for variables that showed normal distribution. The Kruskal-Wallis test was used for comparison of continuous variables that did not show normal distribution. Differences in proportions were compared by the χ^2^ test or Fischer’s exact test where appropriate. All patients with SMR and FMR were divided into two groups, depending on the time period when MV TEER was performed (2010–2015 vs. 2016–2021). This was used for determination of potential trend differences in demographic and clinical characteristics among SMR and FMR patients. Troponin T and NT-pro-BNP initially did not show normal distribution and therefore logarithmic transformation was performed. The *p*-value < 0.05 was considered statistically significant.

## 3. Results

**Figure 1:**
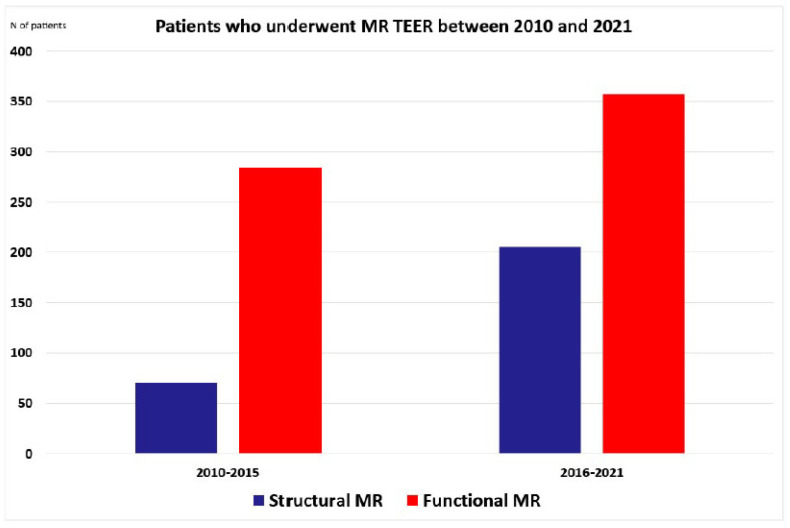
Patients who underwent MR TEER between 2010 and 2021. MR—mitral regurgitation, N—number, TEER—transcatheter edge-to-edge repair.

The study included 917 patients who underwent MV TEER at our institution between January 2010 and March 2021, i.e., 354 patients with SMR (39%) and 563 patients with FMR (61%) (Figure 1). The number of SMR and FMR patients increased over the observed period of time. There was no difference in age, sex distribution or BMI between SMR and FMR patients (Table 1). Systolic blood pressure was similar between the two observed groups, but diastolic blood pressure was higher in SMR patients. The prevalence of hypertension and dyslipidemia was similar between two observed groups, whereas diabetes was more prevalent in patients with FMR (Table 1). NYHA class III and IV were equally distributed between two groups.

Comorbidities such as coronary artery disease, peripheral artery disease, pulmonary hypertension, renal and hepatic failure, as well as previous oncological disease, were similarly present in both groups (Table 1). The same results were obtained for previous interventions such as bypass cardiac surgery, mitral and aortic valve surgery, and TAVR. Patients with FMR had borderline higher percentage of coronary interventions (Table 1). Regarding implantable cardiac devices, there was no difference in prevalence of pacemakers and CRT between groups, but FMR patients had significantly higher percentage of ICD devices (Table 1). EuroSCORE II was significantly higher in FMR than in SMR patients (Table 1).

**Figure 2:**
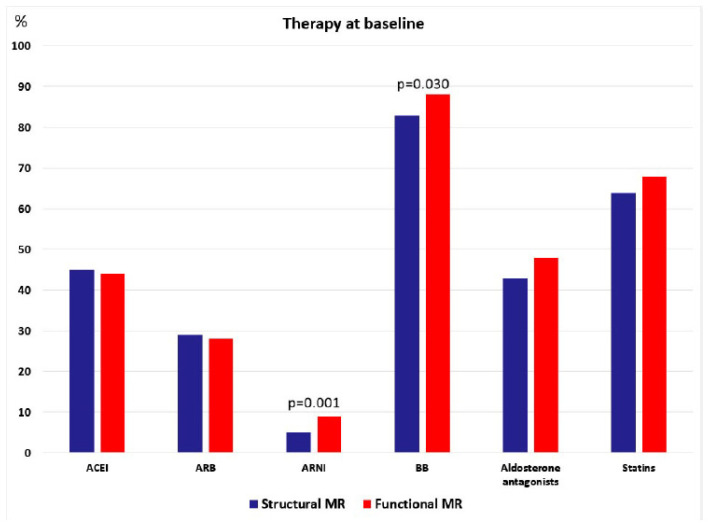
Therapy at baseline.

There was no difference in the prevalence of ACEI, ARB, aldosterone antagonist and statin prescriptions between SMR and FMR patients (Table 1), but ARNI and beta-blockers were more prevalently prescribed to FMR patients (Figure 2). No difference was found in laboratory parameters of kidney function and NT-pro-BNP.

**Figure 3:**
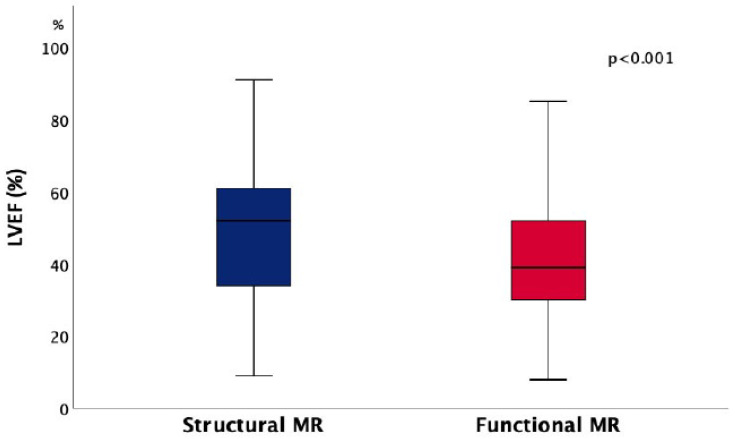
Differences in left ventricular ejection fraction (LVEF) between patients with structural and functional mitral regurgitation (MR).

Echocardiographic examination revealed significantly higher LV diameters in FMR patients, whereas LVEF was significantly lower in these subjects (Table 1, Figure 3). No significant difference was found in LA diameter and interventricular septum thickness.

Intraprocedural results showed no difference in MR severity after TEER in patients with SMR and FMR, separately (Table 1). The risk of mitral stenosis after TEER was similar in both groups (Table 1).

## 4. Hemodynamic Measurements

Our findings showed no significant difference in atrial or ventricular pressures between SMR and FMR patients (Table 2). There was also no difference in pulmonary systolic, diastolic and mean pressures. Mean pulmonary capillary wedge pressure was similar between the two observed groups. Systolic, diastolic and mean systemic blood pressures were similar. Systemic vascular resistance was higher in patients with SMR, whereas no significant difference was observed in pulmonary vascular resistance (Table 2). Cardiac output and cardiac index were similar between SMR and FMR patients (Table 2). There was no significant difference in oxygen saturation between two groups of patients.

### Trend Differences in Different Period (2010–2015 vs. 2016–2021)

The increase in prevalence of SMR patients who underwent TEER procedures between 2010 and 2015 or between 2016 and 2021 (70 vs. 284 patients) was significantly higher than in FMR patients (206 vs. 357 patients, *p* < 0.001) (Table 1). Age, BMI and sex distribution were similar in SMR and FMR patients in different time periods (2010–2015 and 2016–2021) (Table 3). Systolic and diastolic blood pressures were similar in SMR patients in both time periods, but systolic blood pressure was higher in FMR patients who underwent TEER in the period between 2016 and 2021.

**Figure 4:**
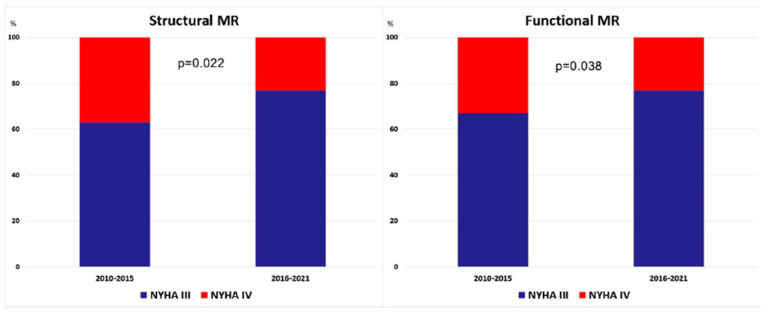
The difference in prevalence of patients with New York Heart Association (NYHA) class III and IV in patients with functional and structural mitral regurgitation (MR) between two periods (2010–2015 and 2016–2021).

Patients with more pronounced symptoms of heart failure and NYHA class IV were more prevalent in the first time period between 2010 and 2015, whereas patients with NYHA class III were more prevalent in the later period (Table 3, Figure 4).

The differences were not noticed in the prevalence of coronary interventions, cardiac surgeries (bypass or valvular surgery) or interventional aortic valve replacement between patients who underwent MV TEER in the period 2010–2015 and 2016–2021 in both groups, SMR and FMR (Table 3).

The majority of comorbidities (hypertension, diabetes, coronary artery disease, dyslipidemia, COPD, asthma, hepatic cirrhosis, previous oncological disease) were equally distributed between SMR and FMR patients in the two different time periods (Table 3). Peptic ulcer was more prevalent among SMR patients who underwent interventional procedure in period between 2010 and 2015, whereas renal failure was more prevalent in FMR patients in the same period of time (Table 3).

Regarding antiarrhythmic devices, pacemaker and CRT were equally present in SMR and FMR patients in both time periods, whereas ICD was more prevalent in FMR patients in the period between 2010 and 2015 (Table 3). EuroSCORE II was significantly higher in SMR patients in the first period of time (Table 3).

Therapeutic approach also changed over the last decade, because ACEIs were predominantly used in the first half of this period, particularly in FMR patients, whereas ARBs were dominant in the last 5 years, particularly in SMR patients, but also in the FMR group, with borderline statistical significance (Table 3). The use of beta-blockers, aldosterone antagonists and statins did not change over the last decade in both groups of patients (SMR and FMR). It should be also noticed that a new group of drug–ARNI was introduced in the last three years and significant portion of FMR patients was switched from ARB to this drug.

Laboratory analyses did not reveal differences in renal function, as well as in NT-pro-BNP levels (Table 3). Regarding echocardiographic findings, LVEF were similar between the early and late period in both groups of patients (SMR and FMR). LV diameters and interventricular septum thickness were similar between early and late period among patients with SMR (Table 3). LA diameter was higher in patients who underwent MV TEER in the first half of the observed period (2010–2015). Among FMR patients, the difference was found only in LV end-diastolic diameter that was also higher in the first half of the last decade (Table 3). Other echocardiographic parameters of LV structure and systolic function in FMR patients were similar between early and late period.

Intraprocedural results revealed similar prevalence in patients with structural and functional MR with different MR severity after TEER between first and second 5 years (Table 1). The risk of mitral stenosis after TEER did not change in period 2010–2015 and 2016–2021 among patients with SMR and FMR (Table 1).

## 5. Discussion

Our study revealed several important findings that warrant further discussion: (i) a considerable number of SMR patients is treated MV TEER and their percentage significantly increased over the last 5 years; (ii) there was no significant difference in demographic and clinical characteristics among SMR and FMR patients who were treated over the last decade with this method; (iii) LV remodeling and systolic dysfunction were more pronounced in FMR patients; (iv) systemic vascular resistance was the only invasive hemodynamic parameter that differed between SMR and FMR patients; (v) operative risk of SMR and FMR patients was significantly higher in the first five years; (vi) medical therapy has also changed over the last decade in MR patients in terms of increased use of ARB and introduction of ARNI.

The number of patients treated with MV TEER significantly increased over the last few years, but our study showed that in Germany and probably the rest of Europe, the trend is going toward higher increase in the number of patients with SMR than FMR–opposite to what is observed in the US. This is an interesting finding because in Europe MV TEER was primarily intended for high-risk FMR patients who would be rejected from open heart surgery. It seems that Europe adopted this approach for both groups of patients because of encouraging results regarding better outcome and reduction of hospital admission in both groups of patients. On the other side, US remained to use an interventional approach only in SMR patients until 2019 and only recently gave approval for FMR patients.

Our findings showed no significant difference in demographic and clinical characteristics between SMR and FMR patients who underwent MitraClip or PASCAL procedure in the last 10 years. The only difference was a higher prevalence of coronary interventions and diabetes in FMR patients. Data on this topic are still conflicting. Öztürk et al. found no difference in demographic and clinical features between SMR and FMR patients [10], whereas Demir et al. found significant higher prevalence of all cardiovascular risk factors and comorbidities in FMR patients [11]. Buzzatti et al. reported higher prevalence of coronary artery disease and CRT in FMR patients, whereas other comorbidities were equally distributed among patients with SMR and FMR [12]. Polimeni et al. revealed higher prevalence of diabetes, prior revascularization and myocardial infarction in FMR patients, while there was no significant difference in other concomitant diseases [13]. Meta-analyses that included 2351 patients showed that diabetes, previous myocardial infarction, renal insufficiency, coronary artery bypass grafting, percutaneous coronary intervention and cardiac resynchronization therapy were more prevalent among FMR patients [14]. Another meta-analysis that involved 2615 patients reported that diabetes, atrial fibrillation, chronic kidney disease, coronary artery disease and chronic obstructive pulmonary disease were more frequently detected in FMR patients [15]. However, it should be emphasized that both meta-analysis included the early period of MitraClip implantation (until then end of 2017) [14,15]. Our study included 917 MR patients from a single center and revealed no difference in comorbidities and cardiovascular risk factors between patients who were treated until 2015 and those who were treated in the last 5 years. However, our results also revealed that EuroSCORE II is significantly lower in SMR in the last 5 years than it was at the beginning, whereas there is no difference in trend of score changing among FMR patients. The present findings also revealed significantly higher prevalence of NYHA class III patients and lower prevalence of NYHA class IV patients in the last 5 years than in was until 2015. This shows that we currently treat MR patients at earlier stage as previously and this particularly refers to SMR patients who are more frequently treated with interventional approach in the last five years. This trend was confirmed in our previous study that divided all patients in quintiles and confirmed continuous increase in SMR patients over last 10 years [16].

Medical treatment in SMR and FMR patients differs only in higher prevalence of ARNI and beta-blocker usage in FMR patients, which is reasonable considering the different underlying etiologies and underlying mechanisms, as well as significantly higher prevalence of heart failure among FMR patients. The trend of shifting ACEIs toward ARBs and introduction of ARNI marked the last five years MV TEER and it was not reported previously. This is not the result of changing the paradigm of medical treatment of MR, but novel approach in therapy of heart failure that involved ARNI, the combination of valsartan (ARB) and neprilysin inhibitor. ARBs (particularly valsartan) replaced ACEIs in therapy of many patients with heart failure because they were prepared for ARNI as the next step of therapy.

The baseline left ventricular structural and functional remodeling was more pronounced in FMR patients, as expected. The similar findings were previously reported [9,10,11,12]. Our results showed that this aspect did not change over the observed period. Laboratory findings regarding renal function and level of NT-pro-BNP were not significantly different between SMR and FMR patients and they remained unchanged over the whole decade.

Baseline invasive hemodynamic parameters were similar between SMR and FMR patients except systemic vascular resistance that was significantly higher in SMR patients. This is the first study that provided detailed hemodynamic assessment of the large number of patients with FMR and SMR who underwent MV TEER. The initial studies included a very limited number of patients and did not find differences in mean pulmonary artery pressure, pulmonary capillary wedge pressure and cardiac index between SMR and FMR [17]. Other investigations showed significantly higher pulmonary pressures and wedge pressure in symptomatic in comparison with asymptomatic SMR patients [18].

There are several important clinical implications of our study. There are no differences in demographic, clinical and hemodynamic parameters between SMR and FMR patients, even though FMR are at higher operative risk mainly due to worse LVEF. Therefore, there is no clinically justifiable reason for not treating any of these groups of patients with this interventional technique. The increased number of SMR patients who are successfully treated with MV TEER at our institution over the last five years might be the reason for full adoption of this interventional technique in all MR patients with inacceptable high risk for surgery, which is approximately half of all MR patients.

## 6. Limitations

Our study has several limitations that should be considered. First, the results were obtained from our registry, and therefore, not all data were available for all patients. This primarily refers to hemodynamic data. However, this was a large, real-world population of patients with SMR and FMR who underwent MV TEER over a period of 11 years, which provided the unique opportunity to perceive trends and changes in this field. Second, this was a single-center study, which made it susceptible to the usual types of biases ascribed to this design, including potential limitation to generalize our findings. Third, patients with previous cardiac surgery due to coronary artery or valvular diseases were not excluded, which might have influenced the final results. Nevertheless, this was a real-world population of patients that we treated in clinical practice, and we believe that this simultaneously represents an important strength of this study. The latest drugs used in the treatment of heart failure patients, such as sodium-glucose co-transporter-2 (SGLT2) inhibitors, were not included in this study as they were first approved only for diabetic patients and significantly after ARNI for heart failure patients. Nevertheless, a limited number of patients with MR take SGLT2 inhibitors, and this would not have significantly changed the final results.

## 7. Conclusions

This is a single-center study that included a large cohort of SMR and FMR patients who underwent MV TEER in the period between 2010 and 2021. Our findings showed no significant difference in demographical, clinical and hemodynamic characteristics between SMR and FMR patients. There was also no significant difference in these parameters among SMR and FMR patients in the first and second half of the last decade. A trend of constantly increasing numbers of treated patients, as well as an increase in the proportion of SMR patients, was noticed in the last five years. Our results might allow an even broader acceptance of this technique and increased treatment volume of high-risk patients with both types of MR.

## Figures and Tables

**Table 1:** Demographic characteristics and clinical parameters of study population.

	Structural MR (n = 354)	Functional MR (n = 563)	*p*
Age (years)	78 ± 9	77 ± 8	0.097
Male (%)	154 (44)	230 (41)	0.450
BMI (kg/m^2^)	25.7 ± 4.4	26.0 ± 5.0	0.461
Systolic blood pressure (mmHg)	126 ± 21	125 ± 20	0.162
Diastolic blood pressure (mmHg)	73 ± 11	71 ± 12	0.016
NYHA class			0.716
III (%)	262 (74)	414 (73)	
IV (%)	92 (26)	149 (27)	
Interventions and surgeries			
PCI (%)	148 (42)	273 (48)	0.049
CABG (%)	54 (15)	96 (17)	0.588
Mitral valve surgery (%)	3 (1)	3 (0.5)	0.681
TAVR (%)	21 (6)	38 (7)	0.680
Aortic valve surgery (%)	21 (6)	29 (5)	0.601
Comorbidities			
CAD (%)	225 (64)	385 (68)	0.110
Previous MI (%)	76 (21)	142 (25)	0.355
Hypertension (%)	286 (81)	455 (81)	1.00
Dyslipidemia (%)	208 (59)	335 (60)	0.836
Diabetes (%)	86 (24)	175 (31)	0.029
Atrial fibrillation (%)	215 (61)	376 (67)	0.064
Peripheral artery disease (%)	25 (7)	56 (10)	0.152
COPD (%)	38 (11)	70 (12)	0.463
OSAS (%)	18 (5)	36 (6)	0.473
Peptic ulcer disease (%)	7 (2)	13 (2)	0.820
Renal failure (%)	172 (49)	280 (50)	0.786
Acute renal failure (%)	20 (6)	23 (4)	0.336
Hepatic cirrhosis (%)	5 (1)	7 (1)	1.00
Previous cancer (%)	57 (16)	99 (17)	0.357
Antiarrhythmia devices			
CRT (%)	28 (8)	56 (10)	0.408
ICD (%)	30 (8)	93 (16.5)	<0.001
Pacemaker (%)	33 (9)	51 (9)	0.907
Scores			
Euro Score II	7.5 ± 7.3	8.7 ± 8.4	0.024
Therapy			
ACEI (%)	159 (45)	248 (44)	0.785
ARB (%)	101 (29)	157 (28)	0.821
ARNI (%)	17 (5)	51 (9)	0.001
Beta-blockers (%)	293 (83)	497 (88)	0.030
Aldosterone antagonists (%)	149 (43)	271 (48)	0.089
Statins (%)	227 (64)	384 (68)	0.249
Laboratory			
Creatinine (μmol/L)	129 ± 72	131 ± 69	0.710
GFR (ml/min/1.73 m^2^)	49 ± 20	48 ± 19	0.248
NT-pro-BNP (pg/mL)	5191 ± 6381	5176 ± 6175	0.975
Echocardiography			
LVEF (%)	49 ± 18	41 ± 16	<0.001
LVEDD (mm)	58 ± 11	61 ± 11	0.001
LVESD (mm)	41 ± 13	47 ± 13	<0.001
Interventricular septum thickness (mm)	10.8 ± 2.4	11 ± 3.0	0.692
LA (mm)	55 ± 9	55 ± 9	0.306
Intraprocedural results			
MR severity after TEER			
MR 1+	283 (80)	473 (84)	0.239
MR 2+	60 (17)	79 (14)	
MR 3+	11 (3)	11 (2)	
Mitral stenosis (%)	8 (2.2)	13 (2.3)	1.00

ACEI—angiotensin converting enzyme inhibitor, ARB—angiotensin II receptor blocker, ARNI—angiotensin receptor II blocker—neprilysin inhibitor, BMI—body mass index, CABG—coronary artery bypass grafting, COPD—chronic obstructive pulmonary disease, CRT—cardiac resynchronization therapy, GFR—glomerular filtration rate, ICD—implantable cardiac defibrillators, LA—left atrium, LV—left ventricle, LVEF—left ventricular ejection fraction, LVEDD—left ventricular end-diastolic diameter, LVESD—left ventricular end-systolic diameter, MI—myocardial infarction, MR—mitral regurgitation, OSAS—obstructive sleep-apnea syndrome, PCI—percutaneous coronary artery intervention, TAVR—transcatheter aortic valve replacement, TEER—transcatheter edge-to-edge repair.

**Table 2:** Hemodynamic measurements in study population.

	Structural MR (n = 140)	Functional MR (n = 230)	*p*
Heart rate (beat/min)	72 ± 15	72 ± 16	0.868
Mean RA pressure (mmHg)	11 ± 6	11 ± 7	0.645
Mean RV pressure (mmHg)	21 ± 12	27 ± 14	0.049
Systolic PA pressure (mmHg)	52 ± 16	50 ± 15	0.189
Diastolic PA pressure (mmHg)	20 ± 9	20 ± 11	0.579
Mean PA pressure (mmHg)	35 ± 17	32 ± 11	0.095
Mean PCWP (mmHg)	23 ± 9	22 ± 9	0.303
Mean LA pressure (mmHg)	20 ± 8	19 ± 10	0.392
LV end-systolic pressure (mmHg)	124 ± 31	137 ± 35	0.284
LV end-diastolic pressure (mmHg)	20 ± 7	20 ± 9	0.937
Systolic BP (mmHg)	124 ± 32	127 ± 28	0.282
Diastolic BP (mmHg)	72 ± 18	64 ± 15	0.169
Mean BP (mmHg)	87 ± 21	87 ± 20	0.754
SVR (dynes/seconds/cm^−5^)	2265 ± 1680	1886 ± 1066	0.008
PVR (dynes/seconds/cm^−5^)	300 ± 235	300 ± 264	0.978
Cardiac output (L/min)	3.9 ± 1.2	3.8 ± 1.1	0.490
Cardiac index (L/min/m^2^)	2.1 ± 0.6	2.1 ± 0.5	0.672
Oxygen saturation in aorta (%)	90 ± 4	91 ± 4	0.270
Oxygen saturation in PA (%)	57 ± 10	57 ± 9	0.545

BP—blood pressure, LA—left atrial, LV—left ventricular, MR—mitral regurgitation, PA—pulmonary artery, PCWP—pulmonary capillary wedge pressure, PVR—pulmonary vascular resistance, SVR—systemic vascular resistance.

**Table 3:** Demographic characteristics and clinical parameters in period 2010–2015 and 2016–2021.

	2010–2015	2016–2021		2010–2015	2016–2021	
	Structural MR (n = 70)	Structural MR (n = 284)	*p*	Functional MR (n = 206)	Functional MR (n = 357)	*p*
Age (years)	77 ± 9	78 ± 8	0.723	77 ± 9	77 ± 8	0.845
Male (%)	25 (36)	129 (34)	0.178	79 (38)	151 (42)	0.374
BMI (kg/m^2^)	25.5 ± 4.1	25.8 ± 4.5	0.692	25.7 ± 4.5	26.1 ± 5.2	0.346
Systolic blood pressure (mmHg)	123 ± 20	127 ± 22	0.166	122 ± 20	126 ± 19	0.042
Diastolic blood pressure (mmHg)	72 ± 11	74 ± 11	0.141	70 ± 11	72 ± 12	0.088
NYHA class						
III (%)	44 (63)	218 (77)	0.022	139 (67)	274 (77)	0.038
IV (%)	26 (37)	66 (23)		67 (33)	82 (23)	
Interventions and surgeries						
PCI (%)	24 (34)	124 (44)	0.177	94 (46)	179 (50)	0.336
CABG (%)	16 (23)	38 (13)	0.062	38 (18)	58 (16)	0.447
Mitral valve surgery (%)	1 (1)	2 (1)	0.485	2 (0.1)	1 (0.2)	0.558
TAVR (%)	3 (4)	18 (6)	0.777	10 (5)	28 (8)	0.222
Aortic valve surgery (%)	5 (7)	16 (6)	0.580	14 (7)	15 (4)	0.179
Comorbidities						
Previous MI (%)	14 (20)	62 (22)	0.439	47 (23)	95 (27)	0.361
CAD (%)	49 (70)	176 (62)	0.208	151 (73)	234 (66)	0.070
Hypertension (%)	59 (84)	227 (80)	0.499	171 (83)	284 (80)	0.374
Dyslipidemia (%)	39 (56)	169 (60)	0.589	121 (59)	214 (60)	0.790
Diabetes (%)	20 (29)	66 (23)	0.354	60 (29)	115 (32)	0.508
Atrial fibrillation (%)	49 (70)	166 (58)	0.101	139 (67)	237 (66)	0.779
Peripheral artery disease (%)	7 (10)	18 (6)	0.299	20 (10)	36 (10)	1.00
COPD (%)	7 (10)	31 (11)	1.00	27 (13)	43 (12)	0.791
OSAS (%)	3 (4)	15 (5)	1.00	13 (6)	23 (6)	1.00
Peptic ulcer disease (%)	4 (6)	3 (1)	0.031	8 (4)	5 (1)	0.079
Renal failure (%)	37 (53)	135 (48)	0.505	117 (57)	163 (46)	0.011
Acute renal failure (%)	1 (1)	19 (7)	0.143	7 (3)	16 (4)	0.660
Hepatic cirrhosis (%)	2 (3)	3 (10)	0.257	1 (0.4)	6 (2)	0.432
Previous cancer (%)	10 (14)	47 (17)	0.720	36 (17)	62 (17)	0.974
Antiarrhythmia devices						
CRT (%)	6 (8)	22 (8)	0.806	21 (10)	33 (9)	0.767
ICD (%)	10 (14)	20 (7)	0.058	45 (22)	48 (13)	0.013
Pacemaker (%)	8 (11)	25 (9)	0.494	22 (11)	29 (8)	0.361
Scores						
Euro Score II	10.4 ± 9.9	6.8 ± 6.3	<0.001	9.5 ± 8.3	8.3 ± 8.4	0.112
Therapy						
ACEI (%)	38 (54)	121 (43)	0.107	112 (54)	136 (38)	<0.001
ARB (%)	10 (14)	91 (32)	0.003	48 (23)	109 (31)	0.079
ARNI (%)	-	17 (6)	-	-	51 (14.3)	-
Beta-blockers (%)	57 (81)	236 (83)	0.723	182 (88)	315 (88)	1.00
Aldosterone antagonists (%)	24 (34)	125 (44)	0.140	93 (45)	178 (50)	0.294
Statins (%)	36 (51)	191 (67)	0.017	139 (67)	245 (69)	0.779
Laboratory						
Creatinine (μmol/L)	127 ± 54	130 ± 76	0.759	131 ± 64	131 ± 71	0.962
GFR (mL/min/1.73 m^2^)	50 ± 18	49 ± 21	0.884	47 ± 19	48 ± 19	0.676
NT-pro-BNP (pg/mL)	4549 ± 5158	5270 ± 6520	0.554	5892 ± 6509	4923 ± 6042	0.145
Echocardiography						
LVEF (%)	50 ± 17	49 ± 18	0.721	43 ± 17	40 ± 15	0.096
LVEDD (mm)	57 ± 10	58 ± 11	0.714	62 ± 12	60 ± 11	0.032
LVESD (mm)	39 ± 12	42 ± 13	0.245	48 ± 15	47 ± 13	0.225
Interventricular septum thickness (mm)	10.9 ± 2.7	10.8 ± 2.3	0.944	10.6 ± 2.3	11.2 ± 3.3	0.287
LA (mm)	58 ± 12	54 ± 9	0.012	56 ± 9	55 ± 10	0.653
Intraprocedural results						
MR severity after TEER						
MR 1+	53 (76)	230 (81)	0.326	165 (80)	308 (86)	0.126
MR 2+	13 (18)	47 (17)		35 (17)	44 (12)	
MR 3+	4 (6)	7 (2)		6 (3)	5 (1)	
Mitral stenosis (%)	3 (4)	5 (2)	0.196	7 (3)	6 (2)	0.245

ACEI—angiotensin converting enzyme inhibitor, ARB—angiotensin II receptor blocker, ARNI—angiotensin receptor II blocker—neprilysin inhibitor, BMI—body mass index, CABG—coronary artery bypass grafting, COPD—chronic obstructive pulmonary disease, CRT—cardiac resynchronization therapy, GFR—glomerular filtration rate, ICD—implantable cardiac defibrillators, LA—left atrium, LV—left ventricle, LVEF—left ventricular ejection fraction, LVEDD—left ventricular end-diastolic diameter, LVESD—left ventricular end-systolic diameter, MI—myocardial infarction, MR—mitral regurgitation, OSAS—obstructive sleep-apnea syndrome, PCI—percutaneous coronary artery intervention, TAVR—transcatheter aortic valve replacement, TEER—transcatheter edge-to-edge repair.

## Data Availability

All data relevant to the study are included in the article.
